# RAPD-PCR reveals genetic polymorphism among *Leishmania major* strains from Tunisian patients

**DOI:** 10.1186/s12879-015-1010-0

**Published:** 2015-07-14

**Authors:** Rihab Yazidi, Jihene Bettaieb, Wissem Ghawar, Kaouther Jaouadi, Sana Châabane, Amor Zaatour, Afif Ben Salah

**Affiliations:** Department of Medical Epidemiology, Laboratory of Transmission, Control and Immunobiology of Infections (LR11IPT02), Pasteur Institute of Tunis, 13 Place Pasteur BP-74, Tunis, Belvedere 1002 Tunisia

**Keywords:** *Leishmania major*, Genetic Polymorphism, RAPD**-**PCR, Tunisia

## Abstract

**Background:**

Zoonotic cutaneous leishmaniasis caused by *Leishmania* (*L.*) *major* is endemoepidemic in the Center and South of Tunisia. The clinical course of the disease varies widely among different patients and geographic regions. Although genetic diversity in *L. major* parasites has been suggested as a potential factor influencing their pathogenic variability, little information on genetic polymorphism among *L. major* strains is available in the literature. This work aimed to estimate the genetic variability within different isolates of *L. major*.

**Methods:**

Our sample comprised 39 isolates (confirmed as *L. major* by restriction fragment length polymorphism typing) from patients experiencing the same clinical manifestations but living in different regions of Tunisia where *L. major* is endemic. Random amplified polymorphic DNA (RAPD) PCR marker polymorphism was estimated by calculating Nei and Li’s genetic distances and by an analysis of molecular variance (AMOVA).

**Results:**

Analysis of the genetic diversity among the isolates revealed a high level of polymorphism (43 %) among them. AMOVA indicated that the highest variability (99 %) existed within the study regions.

**Conclusions:**

Our results revealed a heterogeneous genetic profile for *L. major* with similar clinical manifestations occurring within the different geographical regions. Additional *L. major* isolates from patients, insect vectors, and reservoir hosts from different endemic foci should be collected for further analysis.

## Background

Zoonotic cutaneous leishmaniasis (ZCL) caused by *L. major* is a vector borne disease. In Tunisia, the disease is distributed mainly in the central and southern parts of the country where it is considered a major public health problem.

Various clinical presentations have been reported in patients, varying from asymptomatic infections to ulcerative lesions of the skin, which last for many months and may cause disfigurement and stigma. The clinical pleomorphism of ZCL is associated with multiple factors such as variability in the species or subspecies of *Leishmania* [1], host genetic factors or immune status [1], host-parasite interactions [2], factors related to environmental upheaval depending on the distribution and density of the respective vectors and reservoirs hosts, transmission pressure, parasite density [3] and the presence of other pathogens [4]. However, because all *Leishmania* parasites (including *L. major*) are vector-borne obligate endoparasites, adaptation to the internal microenvironment of their long-term residence in the macrophages of their mammalian reservoir hosts, is considered the most significant factor influencing the evolution of this genus [5]. Several studies have been conducted to identify and type naturally occurring *Leishmania* parasites to attempt to correlate their genetic diversity with their clinical features such as virulence, pathogenicity and drug resistance. This information is crucial for showing the biology of this parasite [6–8].

The present study aimed to investigate genetic variability among *L. major* isolates from patients who displayed *similar clinical features. To achieve this we* used the genetic markers generated by the random amplified polymorphic DNA (RAPD) technique.

## Methods

### Parasite strains and DNA extraction

In total, 39 isolates were used in this study. Isolates were collected between 2007 and 2009 from areas in central and southern Tunisia where ZCL is endemic. Each isolate was obtained from a different human subject who had a typical nodulo-ulcerative skin lesion that had healed within few months of appearance after using only local care. Only individuals aged between 5 and 65 years who gave their written informed consent (or their parents or legal guardians consent in case of minors) were enrolled.

The *Leishmania* isolates were divided into five geographical groups comprising those from the Sidi Bouzid Governorate, the Kairouan Governorate and the Gafsa Governorate, and represented by the three delegations, M’dhila, Metlaoui and Gafsa Center (Table 1). Species identification of all the isolates as *L. major* was achieved by restriction fragment length polymorphism (RFLP) analysis. Isoenzyme typing of one isolate (MHOM/TN/2009/S566) was conducted at the *Leishmania* Reference Center of Montpellier, France. This isolate exhibited the MON-25 zymodeme, the most common zymodeme of *L. major* in Tunisia [9] and was considered a *L. major* reference strain.

**Table 1:** Selection of Tunisian isolates included in the study

Number	Laboratory code	Place of isolation	Disease	Source	RFLP	Species
1	S004	M’dhila	CL	Human	*+*	*L. major*
2	S006	M’dhila	CL	Human	*+*	*L. major*
3	S007	M’dhila	CL	Human	*+*	*L. major*
4	S011	Metlaoui	CL	Human	*+*	*L. major*
5	S015	Metlaoui	CL	Human	*+*	*L. major*
6	S016	Metlaoui	CL	Human	*+*	*L. major*
7	S022	Metlaoui	CL	Human	*+*	*L. major*
8	S025	Metlaoui	CL	Human	*+*	*L. major*
9	S025	Gafsa	CL	Human	*+*	*L. major*
10	S027	Gafsa	CL	Human	*+*	*L. major*
11	S028	Kairouan	CL	Human	*+*	*L. major*
12	S037	Sidi Bouzid	CL	Human	*+*	*L. major*
13	S039	Sidi Bouzid	CL	Human	*+*	*L. major*
14	S050	Sidi Bouzid	CL	Human	*+*	*L. major*
15	S051	Metlaoui	CL	Human	*+*	*L. major*
16	S058	Metlaoui	CL	Human	*+*	*L. major*
17	S060	Mdhila	CL	Human	*+*	*L. major*
18	S065	Mdhila	CL	Human	*+*	*L. major*
19	S066	Metlaoui	CL	Human	*+*	*L. major*
20	S074	Metlaoui	CL	Human	*+*	*L. major*
21	S076	Gafsa	CL	Human	*+*	*L. major*
22	S077	Gafsa	CL	Human	*+*	*L. major*
23	S078	M’dhila	CL	Human	*+*	*L. major*
24	S079	M’dhila	CL	Human	*+*	*L. major*
25	S080	M’dhila	CL	Human	*+*	*L. major*
26	S083	M’dhila	CL	Human	*+*	*L. major*
27	S084	Metlaoui	CL	Human	*+*	*L. major*
28	S085	Metlaoui	CL	Human	*+*	*L. major*
29	S090	Sidi Bouzid	CL	Human	*+*	*L. major*
30	S096	Kairouan	CL	Human	*+*	*L. major*
31	S100	Kairouan	CL	Human	*+*	*L. major*
32	S103	Kairouan	CL	Human	*+*	*L. major*
33	S107	M’dhila	CL	Human	*+*	*L. major*
34	S122	Kairouan	CL	Human	*+*	*L. major*
35	S123	M’dhila	CL	Human	*+*	*L. major*
36	S124	Metlaoui	CL	Human	*+*	*L. major*
37	S125	Metlaoui	CL	Human	*+*	*L. major*
38	S433	Metlaoui	CL	Human	*+*	*L. major*
39	MHOM/TN/2009/ S566*	Metlaoui	CL	Human	*+*	*L. major* MON 25

*Isoenzyme typing was done only for the strain S566

CL: cutaneous leishmaniasis; RFLP: restriction fragment length polymorphism

For genomic DNA extraction, promastigotes corresponding to each isolate were harvested on the sixth day of culture in RPMI 1640 medium supplemented with 20 % heat inactivated fetal bovine serum (Invitrogen, Carlsbad, CA, USA), 2 mM glutamine, 100 U/ml penicillin/50 and U/ml streptomycin at 26 °C. A pellet of 2 × 10^8^ parasites of each isolate was stored at −20 °C. DNA was extracted from each pellet using the QIAamp® DNA Mini Kit (QIAGEN, Germany) according to the manufacturer’s instructions. DNA concentrations were estimated by spectrophotometry by reading the absorbance at 260 nm. Scanning spectrophotometry and agarose gel electrophoresis were used to judge the purity and integrity of the DNA.

### RAPD-PCR

The 16 decameric primers (Invitrogen) described previously [10, 11] were used for this study. All primers used were resuspended in TE buffer, stored at −20 °C, and 10 mM (10pmol/ml) working solutions were prepared. The same person did the RAPD screening and amplification using a single protocol.

Four primers yielded patterns that were nondistinct for all of the isolates and were, therefore, excluded from the study. The excluded primers were A1 (CAGGCCCTTC), AB1-12 (CCTTGACGCA), AB1-14 (TTCCCCCGCT) and AB1-18 (CCACAGCAGT). Hence, the results presented herein are from only 12 of the primers (Table 2).

**Table 2:** Nucleotide sequences of primers used in this study

Primers	Nucleotide sequences	% of G/C
A4	AATCGGGCTG	60
A5	AGGGGTCTTG	60
A7	GAAACGGGTG	60
A8	GTGACGTAGG	60
A10	GTGATCGCAG	60
A15	TTCCGAACCC	60
AB0-01	CCGTCGGTAG	70
AB1-07	GGTGACGCAG	70
AB1-09	TGGGGGACTC	70
AB1-15	GGAGGGTGTT	60
327	ATACGGCGTC	60
329	GCGAACCTCC	70

The RAPD mixtures were processed in 50 μl reactions containing 0.2 mM of each dNTP, 0.3 μM primer, 1 U of Taq DNA polymerase (Invitrogen), 10 x buffer (supplied with the enzyme; 10 mM Tris-HCl, 1.5 mM MgCl2, 50 mM KCl, pH 8.3), and 20 ng of genomic DNA. Amplification was performed in a thermocycler (PXE 0.5 Thermal Cycler, Thermo Electron Corporation, Waltham, USA) using 1 cycle at 94 °C for 2 min, followed by 35 cycles at 94 °C for 1 min, 36 °C for 1 min, 72 °C for 2 min, and final extension at 72 °C for 10 min. A positive control (containing ADN extracted from *Leishmania* culture reference strains MHOM/TN/2009/S566) and a negative control to detect contamination (containing water and no DNA) were included in each PCR run. The amplified products were analyzed on 2 % agarose gels run in 1 × TBE buffer stained with SYBR Safe (Invitrogen), visualized under UV light, and photographed (GenoView, VWR, France). A 1 kb DNA ladder (Invitrogen) was used as a molecular marker.

### Data analysis

Genetic similarity between the isolates was evaluated through simple association, and Nei and Li (1979) genetic distances were calculated using RAPD Distance 1.04 software [12]. The binary matrix was built pair-wise, and the presence or absence of a RAPD band was scored 1 or 0, respectively [13, 14]. The genetic distance dendrogram was constructed using MVSP software package (Multi Variate Statistical Package 3.1) [15]. Analysis of molecular variance (AMOVA) using WINAMOVA 1.55 [16], with the significance levels of the variance components based on 1000 permutations, was applied to quantify the degree of variation between isolates from the same or different collection sites.

### Ethical clearance

Ethical clearance for this research study was obtained from the ethical committee of the Pasteur Institute of Tunis, Tunisia.

## Results

The genetic variability of 39 *L. major* isolates from five endemic foci for *L. major* (Sidi Bouzid, Kairouan and Gafsa represented by the three delegations, Mdhila, Metlaoui and Gafsa Center) was investigated by RAPD. The consistency of the amplified DNA bands at the same or a different electrophoretic position for *L. major* from the different endemic areas as well as from the same endemic area were analyzed. A total of 53 RAPD fragments were generated whose size ranged from 0.1 to 2.3 kb. The reproducibility of the results was confirmed by repeating the RAPD-PCR analysis with 39 randomly selected isolates. The profile of the isolates in the reproducibility study was similar to their profiles in the original study. The number of reproducible bands produced by each primer ranged from 1 to 10 with an average of 4.5 polymorphic bands per primer.

**Fig. 1:**
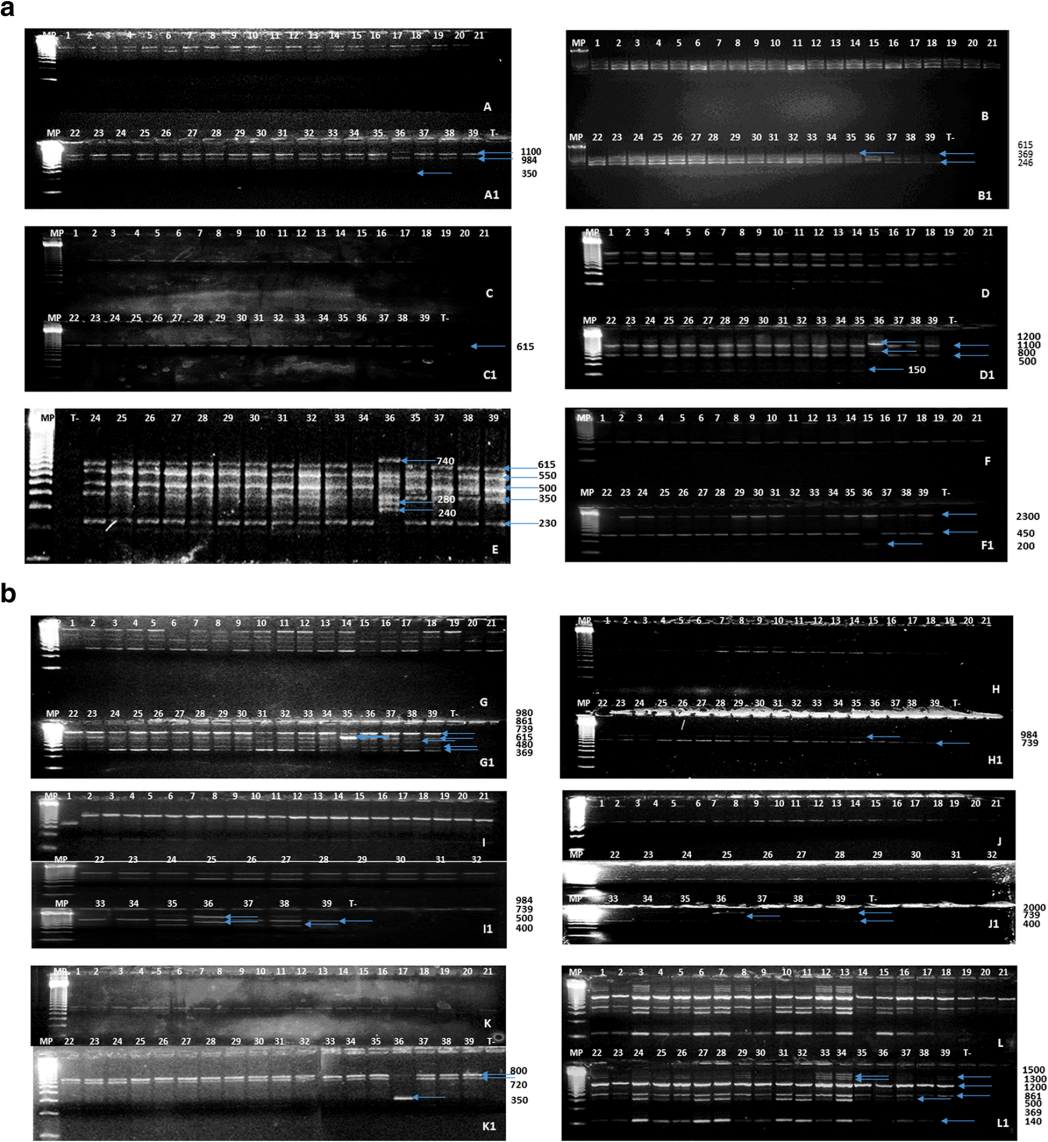
**a and b.** Random amplified polymorphic DNA (RAPD) amplification profiles observed with AB1-09 (A-A1), A4 (B-B1), A5 (C-C1), AB01-07 (D-D1), 329 (E), A7 (F-F1), 327 (G-G1), AB1-15 (H-H1), A10 (I-I1), A15 (J-J1), A8 (K-K1) and AB0-01 (L-L1) primers. Each lane corresponds to a DNA isolate, which is identified by the number reported in Table 1. DNA size markers (1 kb DNA Ladder; mark, Sigma)

The RAPD profiles are illustrated in Figs. 1a and b. The greatest number of bands (10) was produced by primer 329, while the A5 primer produced only one band. The resulting band pattern shows that the S124 isolate from Metlaoui had a completely distinct profile from the other isolates, yielding two major products of 0.1–2.3 kb, thereby differentiating it from *L. major* DNA.

The polymorphic band percentages ranged from 16 to a maximum of 68 with an average polymorphism level of 43 %. The polymorphism levels varied according to the primers used and the geographic origin of the isolates (Table 3). The most polymorphic RAPD markers were identified with AB01-01 and 329 primers with a percentage of 68 %, while the AB1-09 primer produced the lowest polymorphism (16 %). On average, for all primers, isolates from Metlaoui produced the highest percentage (91 %) of polymorphic bands, while the lowest percentage was seen among the Kairouan isolates (23 %).

**Table 3:** Percentages of polymorphism detected by RAPD among *Leishmania major* isolates

	Geographical origin					
	Sidi-Bouzid	Kairouan	M’dhila	Metlaoui	Gafsa Center	Total
Primers						
A4	0	0	67	100	0	33
329	40	70	90	80	60	68
AB01-07	0	0	0	100	0	20
A7	33	33	33	100	33	46
A10	0	0	100	100	0	40
327	50	50	83	100	33	63
AB01-01	71	43	71	85	71	68
AB1-15	50	50	50	50	50	50
AB1-09	0	0	0	80	0	16
A15	25	0	25	100	25	35
A5	0	0	100	100	0	40
A8	33	33	33	100	33	46
Total	25	23	54	91	25	43

**Fig. 2:**
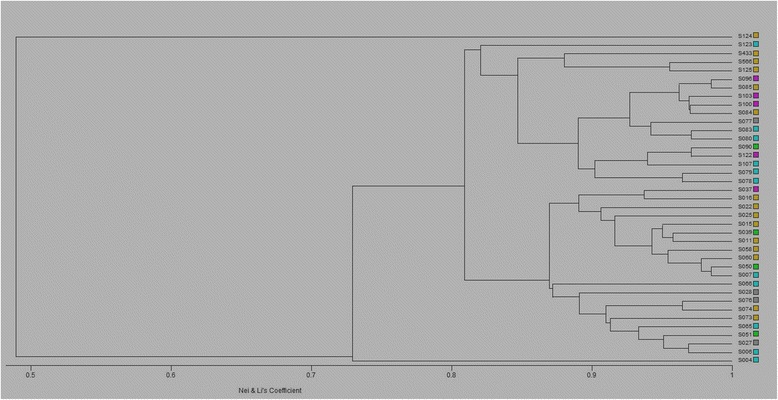
The dendrogram constructed using Nei’s genetic distances for the 39 *L. major* isolates

The genetic identity between the pairs of individuals ranged from 0.59 to 1.00, as based on the criteria of Nei and Li (1979). The dendrogram constructed from Nei’s genetic distances showed two obvious clusters (Fig. 2). The S124 strain belonging to the Metlaoui area appeared on its own, while all the other strains formed a cluster. The major cluster was divided further into two branches with one consisting of the S004 strain from M’dhila while the second divided the remaining strains into several sub-branches. We did not observe any specific clusters that corresponded with the geographical origin of isolates.

The hierarchical analysis of the molecular diversity in the isolates using AMOVA was performed with genetic distances between pairs to analyze the partitioning of RAPD variation in *L. major* isolates among and within populations belonging to the different areas (Table 4).

**Table 4:** Partitioning of genetic variation in *L. major* isolates among and within foci based on AMOVA

Source of variation	df	sum of squares	variance component	% of variance	Φ_ST_
Between foci	5	20.626	0.061	1.1	0.011^*^
Within foci	34	190.193	5.593	98.9	
Total	39	210.820			

^*^ P value not significant

AMOVA analysis within and among the study areas revealed that 98.9 % of the total variation resided between isolates within foci, and only 1.1 % within isolates between foci (Table 4).

Despite the fairly low structuring between foci, the phi-statistics (Φ_ST_) between the population pairs were variable. The largest Φ_ST_ value (0.189) was observed between the Gafsa Center and Kairouan regions, while the lowest value (0.006) was between M’dhila and Kairouan.

## Discussion

ZCL caused by *L. major* in Tunisia has various clinical manifestations. Herein, we used RAPD-PCR to study genetic diversity among 39 *L. major* isolates from the central and southern parts of Tunisia where the parasite is endemic. The twelve polymorphic primers we used revealed a high level of polymorphism (43 %) among the *L. major* isolates collected from patients with similar clinical manifestations of the disease.

Despite many studies suggesting that the genetic variability observed among *L. major* isolates is correlated with different clinical manifestations [6, 7, 17] or responses to antileishmanial treatments [17], our findings suggest that genetic diversity can also be found among *L. major* isolates that cause the same clinical picture. Obviously, the severity of the disease is influenced by the pathogenesis of the parasite species or strain and the host immune responses against it [18]. In fact, a series of complex and not fully understood interactions that occur between species-specific virulence factors and genetically determined cell-mediated immunity contributes to disease susceptibility, as shown in murine models [2, 19–21], but the contribution of each component within a particular region is unknown in humans. Therefore, this raises the question of whether variation in the pathogenicity of the parasite and the clinical pattern of ZCL may be related to genetic heterogeneity in the parasite. However, to address this question it is first necessary to characterize genetic polymorphism in the parasite.

Furthermore, considerable heterogeneity was detected among isolates belonging to the same endemic area, with percentages ranging from 23 to 91 %. In agreement with this result, a previous study in Iran showed substantial variability between some isolates from the same regions using the same RAPD technique [22]. This result might be explained by considerable gene flow among isolates belonging to the same area. Genetic exchange may result from the relatively old evolutionary history of *L. major* in the study sites. Additionally, the zoonotic transmission cycles and the number of reservoir hosts and vectors are known to play an important role in sustaining genetic diversity. Transmission of *L. major* in Tunisia is associated with one major vector (*Phlebotomus papatasi*), but the existence of different species of wild rodent (*Psammomys obesus, Meriones shawi, Meriones libycus*) serving as animal reservoir hosts, and sometimes their coexistence within a small geographic area seems to support this hypothesis [23]. *L. major* strains isolated from both sandflies and rodents should be analyzed for a reliable characterization of *L. major* Tunisian strains.

Although our study isolates from within the same area exhibited genetic polymorphism, lower genetic diversity was found between populations of *L. major* based on their collection sites. This was first noted in the dendrogram built from the RAPD-PCR data based on Nei’s genetic distances. The dendrogram showed no significant clustering of the isolates according to their geographical origin, a finding that was confirmed later by the AMOVA analysis, which indicated that only 1.1 % of the observed variation occurred among the groups, while 98.9 % occurred among isolates within the groups. This finding most likely reflects the unbalanced and low number of isolates per area sampled rather than a lack of correlation between genetic polymorphism and the geographical origin of the parasite isolates, as has been noted before [24]. Investigating this type of correlation was beyond the scope of the present work, and should be tested using an appropriate sampling strategy with a larger sample size per group to reveal potential genetic diversity. The underestimation of genetic variability between regions caused by sampling differences was also supported by the fact that the highest polymorphism percentages were obtained within the Metlaoui and M’dhila regions, which included the highest numbers of isolates among the five study regions.

Some authors have failed to establish an association between genetic variability in their isolates and their geographic distribution when too few strains and/or poorly discriminating markers were used, while others explained the lack of focus-specific genotypes by the small size of the country and the frequent migration between foci [25, 26].

However, other studies have identified geographic structuring of *L. major* populations using the RAPD technique [22], or other PCR-based methods targeting different nuclear multi-copy sequences or antigen-coding genes, followed by RFLP, permissively primed intergenic polymorphic-polymerase chain reaction, single-stranded conformation polymorphism analysis, or multilocus microsatellite typing [25, 27, 28]. Geographical differences have been attributed by some authors to the existence of different populations of vectors and/or to different species of rodent hosts [25, 27–29]. Thus, *Leishmania* strains isolated from different stages of the parasite life cycle belonging to the same or different geographic origins need to be investigated to explain the mechanisms involved in the evolution of *L. major*.

Both AB01-01 and 329 primers produced considerable genetic variability between *L. major* isolates from different regions of Tunisia as well as within the same regions. These primers were chosen because of their reproducibility and ability to reveal intraspecies variation.

The RAPD technique was used for identification and differentiation of old world species of *Leishmania* [10, 25, 27–29]. For *L. major*, these authors reported that the RAPD technique amplified a 0.45 kb product with the A7 primer and generated a 2.7–1.9 kb product with the A10 primer. Nevertheless, our results suggest the validity of the RAPD technique when used for the identification and characterization of *Leishmania* species, especially *L. major*. The RAPD technique amplified a 0.2–2.3 kb product with the A7 primer and generated a 0.2–1 kb product with the A10 primer. The resulting band pattern shows that the S124 isolate from Metlaoui produced different amplicons from the primers.

The first case of cutaneous leishmaniasis in Tunisia was described more than 120 years ago [30] in the region of Gafsa. Since this date, this province has been known as an active focus of ZCL. However, in 2012, twenty-four human cases of cutaneous leishmaniasis caused by *L. killicki* were reported for the first time in Metlaoui district [31]. This important set of data about the new *L. killicki* focus in Metlaoui, and the mixed transmission cycle in this province, allows speculation for S124 about the existence of hybrids and gene flow between genetically different populations.

This is the first time that such a large number of this species isolated from humans have been included from different endemic foci in Tunisia. Here, we have used the RAPD technique as a tool to identify genetic variation within *Leishmania* species. Even though our results have shown that DNA fingerprinting and RAPD analysis were able to discriminate between different *L major* isolates of closely related strains, highly polymorphic markers using fast evolving microsatellites need to be developed to achieve the highest discrimination level among strains. According to these markers, microsatellites are hypervariable, genetically neutral, co-dominant and are, therefore, ideal to reliably address the role that genetic polymorphism plays in the epidemiology of *Leishmania* disease at a fine scale [32].

## Conclusions

Our results suggest that isolates of Tunisian *L. major* are highly polymorphic, despite inducing similar clinical manifestations and belonging to the same geographical origin. This finding requires confirmation using a method with a higher discriminatory capacity and with larger sample size to decipher more fully the pathogenesis of the parasite and show the mechanisms by which it circulates in a restricted geographical area. In future research it would also be worth collecting samples from patients, vectors, and reservoir hosts in the main areas where the parasite is endemic to help improve our understanding of the molecular epidemiology of ZCL in Tunisia.

## Abbreviations

ZCL: Zoonotic Cutaneous Leishmaniasis
RAPD: Random amplified polymorphic DNA
AMOVA: Analysis of molecular variance
Φ_ST_: Phi-statistics
